# Cosmetic Interventions for Skin Microbiome Modulation: Current Strategies and Future Directions

**DOI:** 10.1111/srt.70352

**Published:** 2026-04-16

**Authors:** Raquel Taléns‐Visconti, Octavio Diez‐Sales, Amparo Nácher

**Affiliations:** ^1^ Department of Pharmacy and Pharmaceutical Technology and Parasitology Faculty of Pharmacy and Food Sciences University of Valencia Valencia Spain

**Keywords:** cutaneous dysbiosis, inflammatory skin diseases, microbiome–friendly cosmetics, probiotics/prebiotics/postbiotics/paraprobiotics, skin barrier, skin microbiome

## Abstract

**Background:**

Human skin harbors a highly diverse and dynamic microbiome that maintains barrier function and homeostasis, while endogenous/exogenous factors and cosmetic products modulate microbial balance. Dysbiosis contributes to inflammatory diseases like atopic dermatitis and acne; however, evidence for microbiome‐targeted cosmetics remains preliminary. This article critically examines the impact of cosmetic products on the skin microbiota, distinguishing between potentially harmful formulations and those specifically developed to preserve microbial balance, collectively termed “microbiome‐friendly” cosmetics.

**Materials and Methods:**

This narrative review synthesizes clinical, microbiological, and mechanistic studies on the skin microbiome's composition, endogenous/exogenous determinants, dysbiosis in skin diseases, and the impact of conventional and microbiome‑targeted cosmetics. Databases were searched up to January 2026 for peer‑reviewed studies, with emphasis on human clinical trials and systematic analyses.

**Results:**

Most conventional cosmetics do not induce major dysbiosis in healthy skin when properly formulated. For advanced formulations, including ingredients such as probiotics, prebiotics, postbiotics, and paraprobiotics, early clinical and microbiological studies report promising benefits without compromising microbial diversity. However, evidence is limited by heterogeneous designs, small sample sizes, and the lack of standardized criteria for “microbiome‑friendly” claims.

**Conclusions:**

The evidence indicates that informed cosmetic selection and use of skincare products support microbiome balance as a complementary skin health strategy as may be key for both preventative and therapeutic strategies in managing skin disorders, just as allowing a dynamic understanding of the skin microbiome to improve human health.

## 1 Introduction

Human skin, owing to its large surface area, plays a key role as the primary point of direct contact with the environment. Beyond acting as a simple protective barrier, it is colonized by a complex and dynamic community of microorganisms that constitute the skin microbiome. This assemblage is comprised predominantly of bacteria, but also includes fungi, viruses, archaea, and dust mites, which coexist in equilibrium with the host. Far from being detrimental, these microorganisms maintain a symbiotic relationship with the skin and are considered an essential component of the cutaneous ecosystem [1].

The formation of the skin microbiome begins at birth, shaped by several factors including mode of delivery (vaginal or cesarean), neonatal feeding practices, environmental conditions, and early use of antibiotics. Thereafter, it evolves throughout the lifespan, adapting to physiological, hormonal, and environmental changes characteristic of each stage [2].

The structure of the skin microbiome is not uniform across the body; rather, it varies according to body region, depending on factors such as humidity, sebum production, and skin dryness. Each type of area—sebaceous, moist, or dry—harbors distinct microbial communities adapted to their local conditions [3]. Thus, a thorough understanding of how the skin microbiota is shaped and transformed over time is fundamental both for the prevention and the management of various skin disorders [2, 4].

The equilibrium of the skin microbiome is fundamental for the maintenance of cutaneous homeostasis [1]. One of its most important roles is the modulation of the immune system. In fact, commensal microorganisms educate the cutaneous immune system, promoting a tolerant response toward beneficial species and an active response to potential threats [2]. These interactions occur primarily via pattern recognition receptors on skin and immune cells, facilitating the controlled production of cytokines, the activation of resident lymphocytes, and the maintenance of a balanced immune response [4].

In addition, the microbiome acts as a natural barrier against pathogens, competing with them for space and nutrients, and even secreting antimicrobial peptides, such as bacteriocins and fatty acids, that hinder their proliferation. This competitive mechanism constitutes an effective means of protection that reinforces cutaneous defense without resorting to excessive inflammation [5].

Another key aspect is its contribution to the maintenance of barrier function, not only through the stimulation of structural protein production by keratinocytes but also by influencing the chemical environment of the skin, such as pH and sebum production. This regulation of the cutaneous milieu helps to promote the growth of beneficial microorganisms and to limit that of pathogens, thus maintaining a healthy balance [2].

Finally, microbial balance is vital for preserving skin homeostasis. Its stability enables the skin to efficiently respond to environmental factors without developing chronic inflammatory states [1]. Any significant alteration in this community can compromise immunological function, the physical barrier, and the stability of the dermal ecosystem, thus contributing to the development of various skin disorders.

The *objective* of this paper is to analyze the available scientific evidence on the cutaneous microbiome and its relationship with skin health. Moreover, the effect of cosmetic products on the skin microbiota is evaluated including microbiome‐friendly formulations.

## 2 Microbiome Composition: Role in Cutaneous Homeostasis

The human skin microbiome is composed of a wide variety of commensal microorganisms that coexist in balance with the host and fulfill essential functions such as defense against pathogens, regulation of the immune system, maintenance of physiological pH, and integrity of the epidermal barrier [6, 7]. Table 1 synthesizes information about the human skin microbiome, its main microbial genera, and functions.

**TABLE 1:** Main composition of the human skin microbiome.

Microbiota composition	Main Functions	Observations	References
*Cutibacterium (C. acnes)*	Sebum metabolism into short‐chain fatty acids; maintains acidic environment that suppresses pathogens	Major inhabitant of sebaceous sites; usually commensal but some strains can act as opportunists in dysbiosis	[7, 8]
*Staphylococcus (S. epidermidis, S. hominis, S. capitis)*	Produces antimicrobial substances; direct competition with *S. aureus* and other pathogens; immune modulation	Key immune protector; most studied among staph species owing to high abundance and clinical relevance	[7, 8]
*Streptococcus (S. mitis)*	Putative immunomodulatory effect, still under investigation	Less characterized; suggested roles in immune tolerance	[9]
*Corynebacterium*	Maturation of skin barrier; bacterial competition; sweat composition modification	Impacts microbiome composition and body odor	[6]
*Micrococcus*	Potential antioxidant functions	Relative abundance can fluctuate depending on skin niche	[7, 9]
*Pseudomonas*, *Enhydrobacter*	Immunomodulatory association (unclear relevance)	Functional roles remain poorly defined	[9, 10]
*Lactobacillus*, *Bifidobacterium*, *Clostridium*	Associated with immune maturation in skin; more common in early development, sensitive or experimental conditions	Usually known as gut microbes but identified occasionally in cutaneous settings	[7, 8]
Fungi	Contribution to immune regulation and microbial ecosystem stability	Particularly abundant among fungi in skin microbiome	[7, 8]
Mites	Microenvironmental balance and immune regulation	Less studied in humans; contribute to ecosystem equilibrium	[7, 8]

Microbial diversity observed on the skin is largely determined by the structural and functional differences characteristic of each body region. Factors such as the concentration of sebaceous and sweat glands, sebum quantity, degree of moisture, pH, exposure to the external environment, and thickness of the stratum corneum vary among anatomical sites and directly influence the composition, abundance, and variety of the microbiome present in each [3].

**FIGURE 1:**
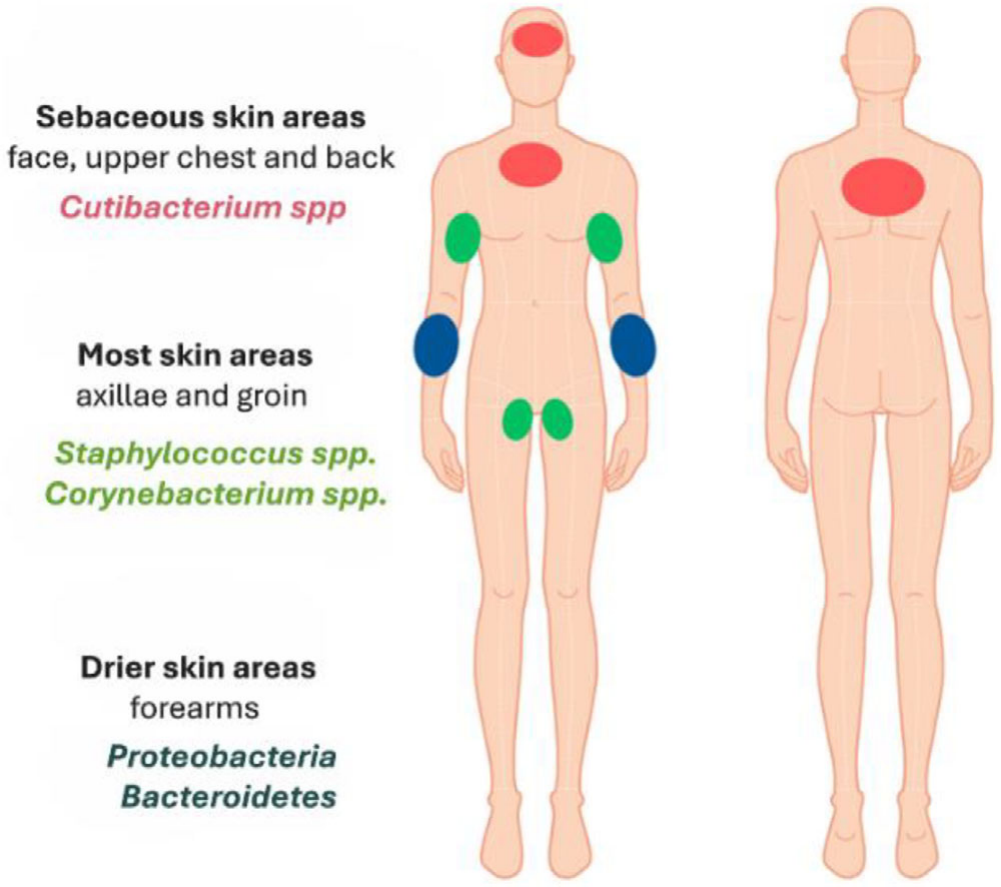
Schematically illustration of the anatomical distribution of the different cutaneous microenvironments and their main associated microbial genera.

From a microbiological perspective, the skin can be divided into three main microenvironment types: sebaceous, moist, and dry (Figure 1). Sebaceous‐rich areas—such as the face, upper chest, and back—tend to harbor lipophilic microorganisms, with *Cutibacterium* spp. being one of the predominant genera. In contrast, more moist areas like the axillae and groin are predominantly colonized by bacteria such as *Staphylococcus* spp. and *Corynebacterium* spp. Lastly, drier skin surfaces, like the forearms, generally have lower sebaceous load and moisture retention, resulting in greater microbial diversity, with phyla such as *Proteobacteria* and *Bacteroidetes* being more abundant [3].

To understand how the composition of the microbiome varies across different body sites, biological diversity metrics such as alpha and beta diversity are employed. Alpha diversity describes how many species coexist within a given skin area and how evenly they are distributed. One of the most common measures used to quantify this is the Shannon index. Conversely, beta diversity compares microbial communities between different body regions or between individuals, thereby helping to detect variations and to describe the complexity of the cutaneous ecosystem [3].

One of the most studied regions in terms of microenvironmental diversity is the face, due to its high exposure and anatomical variability. Recent investigations have demonstrated that even within the same anatomical area, notable microenvironmental variations can be observed, which directly influence the composition of the skin microbiome. For example, regions with high sebaceous activity such as the nose and chin exhibit a microbial profile distinct from that of the lateral cheeks or the eyelids, reflecting differences in lipid secretion, surface moisture, and environmental exposure [9, 11].

Ultimately, understanding this spatial complexity of the cutaneous microbiome is key not only to correctly interpreting microbiological studies but also to designing more precise therapeutic and cosmetic strategies adapted to the characteristics of each skin zone.

### 2.1 Factors Affecting Skin Microbiota

The composition of the skin microbiome is influenced by a complex interplay of factors that directly affect its composition, diversity, stability, and regional distribution across the human body [12]. These factors can be grouped into endogenous and exogenous, each operating through distinct mechanisms upon the microbiota, as summarized in Table 2.

**TABLE 2:** Endogenous and exogenous factors affecting the composition of the skin microbiome.

Factor	Examples	Impact on microbiome
Endogenous or intrinsic	Age, gender, cutaneous phenotype, pH, sebum levels, genetics	Modulate microbial diversity and stability according to life stages, physiological and immunological characteristics
Exogenous or extrinsic	Climate, geographic location, pollution, diet, stress, lifestyle, cosmetics, pharmacological treatments	Alter barrier function, pH, and microbial composition

Understanding these elements enables better interpretation of microbial variations under different skin conditions and guides more accurate dermatological interventions. Nevertheless, it should be noted that the distinction between endogenous and exogenous factors is largely conceptual, as many determinants of the skin microbiota (e.g. stress, diet, cosmetic use, and pharmacological treatments) act through interrelated pathways and often reinforce each other's effects on skin physiology and microbial composition.

#### 2.1.1 Endogenous Factors

Among endogenous factors, intrinsic to the organism, age, gender, cutaneous phenotype, sebum production, skin pH, and genetics are noteworthy [3, 13].

**FIGURE 2:**
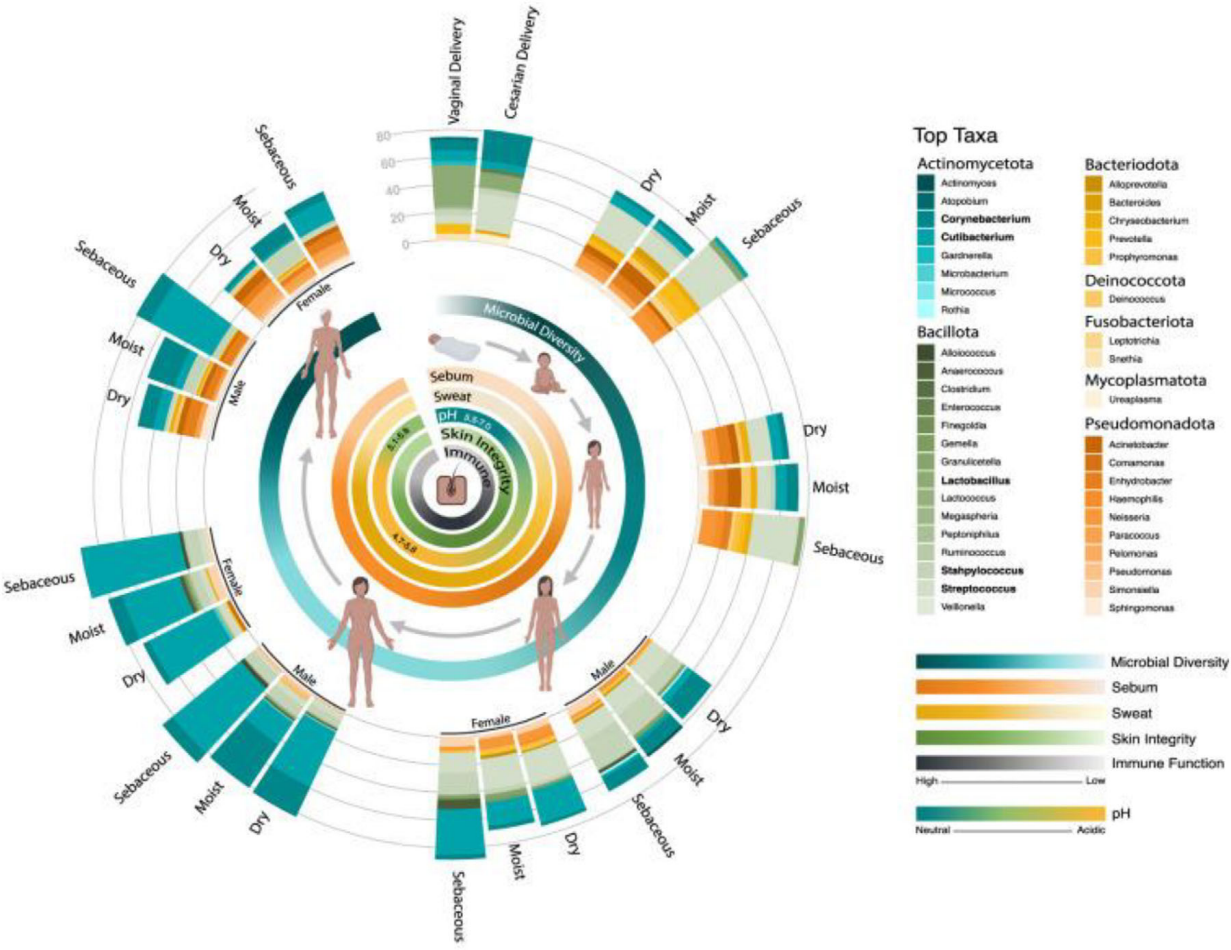
Dynamic interplay between skin and its microbiome throughout life [15]. This is an open access article published by Portland Press Limited on behalf of the Biochemical Society and distributed under the Creative Commons Attribution License 4.0 (CC BY) https://creativecommons.org/licenses/by/4.0/.

The skin microbiome shifts notably throughout life, from the low diversity observed in newborns, to the emergence of lipophilic species with puberty, and typically an increase in overall alpha diversity in older adults due to reduced dominance of protective commensals such as *Cutibacterium acnes* and *Staphylococcus epidermidis*, coupled with higher representation of environmental/transient species [14, 15]. Indeed, it is well‐known that human skin aging is accompanied by microbiota alterations, with both intrinsic and extrinsic factors playing significant roles, such as climate, diet, and genetics that modulate the skin microbiome dynamics [16]. Figure 2 summarizes the evolving interplay of the skin microbiome over the lifespan [15]. Gender is among the most extensively studied aspects. Differences are observed between the microbiomes of men and women, influenced by physiological features and personal care practices [12]. Similarly, the physicochemical properties of the skin, such as hydration, sebum production, and pH, generate specific microenvironments that determine microbial diversity [12] (see Figure 2). A study found that women exhibit higher alpha diversity than men, indicating a richer and more balanced microbiota [3]. This difference is related to physiological features such as a more acidic skin pH, lower sebum and sweat production, and a thinner epidermal barrier. In women, aerobic genera such as *Sphingomonas*, *Pelomonas*, and *Pseudomonas* predominate, whereas in men—with thicker skin and higher sebaceous activity—lipophilic and anaerobic bacteria such as *Cutibacterium* spp. and *Anaerococcus* spp. prevail. This results in a less diverse microbiota dominated by species specialized in oily and low‐oxygen environments [3]. Hormonal activity and age are also closely linked to changes in the cutaneous microbiome. In women, hormonal fluctuations associated with the menstrual cycle influence parameters such as skin hydration, sebum secretion, and transepidermal water loss. It has been observed that women with irregular menstrual cycles exhibit a higher proportion of lipophilic bacteria such as *Cutibacterium* spp. and *Staphylococcus* spp., suggesting that female sex hormones not only regulate physiological functions but also indirectly affect the skin's microbial composition [17]. During adolescence, increased androgenic hormones enhance sebum production, which modifies the lipid milieu of the skin and promotes the growth of certain inflammatory strains of *C. acnes* [18]. Longitudinal data in healthy males from birth to 25 years of age confirm marked age‐dependent shifts in dominant skin taxa, reinforcing the concept of a dynamic, maturation‐linked microbiome rather than a static community [19]. As aging progresses, the skin also undergoes microbial changes. In elderly individuals, different studies demonstrate a net increase in overall alpha diversity despite site‐specific reductions,, especially in areas such as the forearm, possibly due to progressive deterioration of the epidermal barrier and decreased local immune response [13, 15, 16].

Various studies suggest that genetics also influences microbiome composition by affecting factors such as the immune system, structure of the epidermal barrier, or lipid secretion [13]. Mutations in genes involved in epidermal barrier integrity—such as those encoding filaggrin or tight junction proteins—may compromise the skin's protective function and favor colonization by opportunistic microorganisms, contributing to microbial imbalance and increased susceptibility to cutaneous inflammation [20].

#### 2.1.2 Exogenous Factors

The most relevant exogenous influencing the skin microbiome factors include environmental conditions, such as climate and geographic location, lifestyle habits (including stress an diet), the use of cosmetic and hygiene products, as well as topical and systemic pharmacological treatments [3]. These variables directly affect key mechanisms such as perspiration, skin pH, and barrier function, which are crucial for maintaining the balance of the cutaneous ecosystem. Alterations in acidity, for example, can promote dysbiosis [21].

Urbanization and exposure to environmental contaminants have a direct impact on the skin microbiome, especially in exposed areas such as the face. Variables like ultraviolet radiation, temperature, humidity, and atmospheric pollution affect microbial colonization. Compared to rural or natural environments, urban lifestyles are often associated with reduced microbial diversity and higher prevalence of resistant microorganisms, which may be linked to a higher predisposition to inflammatory skin disorders [20, 22, 23].

Moreover, stress impacts the microbiota through neuroendocrine and immunological mechanisms, altering parameters like sebum secretion, hydration, and immune response. Factors such as sleep quality exert an indirect effect on the cutaneous ecosystem [24]. Indeed, adequate sleep regulates key processes, including skin hydration, vasodilation, and regenerative capacity. Sleep deprivation or circadian rhythm disturbances can interfere with these mechanisms and alter the skin's response to external agents. Furthermore, dysregulation of hormones such as melatonin and cortisol may promote oxidative stress, accelerate skin aging, and weaken barrier function, facilitating a microbial disturbance [25]. In addition, increasing recognition is given to the interaction between the gut and skin microbiota, reinforcing the idea that the body functions as an interconnected system. The so‐called gut–skin axis suggests that modifications in intestinal flora could reflect in the skin via immune and neuroendocrine mechanisms, opening new possibilities for systemic approaches to dermatological diseases [18]. Balanced diets, rich in fiber, fruits, vegetables, and healthy fats, promote intestinal microbial diversity—a factor that can positively affect the skin via the gut–skin axis. Conversely, a diet high in refined sugars, trans fats, and ultra‐processed foods may promote microbial imbalances [26].

Finally, the use of pharmacological treatments, particularly topical or systemic antibiotics, can significantly disrupt the microbial balance of the skin [13]. Topical antiseptics, such as 70% ethanol or chlorhexidine, temporarily reduce bacterial load and the commensal microbes diversity, impacting local immunity [27]. Both topical and systemic antibiotics are known to be highly disruptive. In patients with atopic dermatitis, topical mupirocin effectively reduces *S. aureus* but also diminishes microbial diversity [28]. Similarly, systemic minocycline, used in the treatment of acne, reduces *C. acnes* and *Lactobacillus* spp., and simultaneously promotes the overgrowth of opportunists such as *Pseudomonas* and *Streptococcus* [29], evidencing ecological disruption of the skin ecosystem characterized by loss of protective species.

**FIGURE 3:**
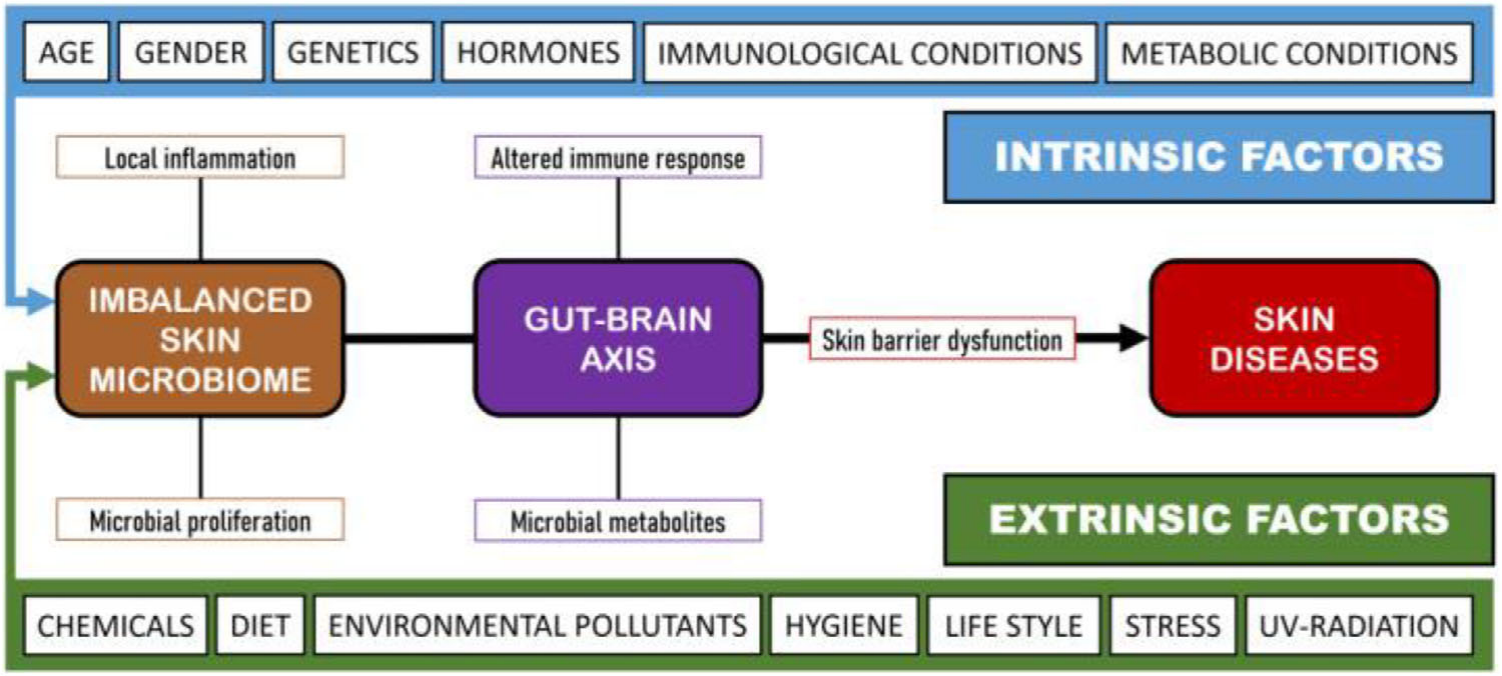
Influence of intrinsic and extrinsic factors on skin diseases [30]. Licensee MDPI, Basel, Switzerland. This article is an open access article distributed under the terms and conditions of the Creative Commons Attribution (CC BY) license (https://creativecommons.org/licenses/by/4.0/). https://creativecommons.org/licenses/by/4.0/.

In summary, understanding both endogenous and exogenous factors under various skin conditions allows for a deeper interpretation of microbial imbalance and supports more precise dermatological interventions. Figure 3 illustrates how these factors may disrupt the balance of the skin microbiome, leading to decreased microbial diversity, increased colonization by opportunistic pathogens, and consequent impairment of skin barrier integrity and heightened susceptibility to inflammatory disorders.

The interplay of endogenous factors shapes a dynamic environment that directly influences the diversity and stability of the cutaneous microbiome, and understanding them is fundamental for the effective management of dermatological disorders. In parallel, exogenous factors can modify the composition and stability of the cutaneous microbiome. Although certain microbial alterations may be transient, chronic or repeated exposure to aggressive agents or antibiotics can lead to persistent dysbiosis, compromise epidermal barrier integrity, and trigger inflammatory responses. Therefore, a more cautious and microbiota‐respecting dermatological approach is recommended.

## 3 Cutaneous Dysbiosis and Its Relationship With Skin Diseases

Dysbiosis refers to an imbalance in the microbiome composition, characterized by a reduction in microbial diversity, shifts in species proportion, and the proliferation of pathogenic or opportunistic microorganisms, all of which exert negative effects on skin health [5]. This phenomenon can compromise cutaneous homeostasis and local immune tolerance, resulting in uncontrolled inflammatory responses, disruption of the stratum corneum, and increased susceptibility to external stimuli. Dysbiosis may thus play a key role in the development and progression of various inflammatory skin diseases, such as psoriasis, atopic dermatitis, and acne [4, 31, 32]. In these cases, dysbiosis is not only a consequence of the disease but also a contributing factor to its onset and progression. In fact, the disruption of microbial balance constitutes a trigger or amplifier of the inflammatory process.

*Atopic dermatitis*, one of the most extensively studied conditions, is a chronic, recurrent inflammatory skin condition primarily characterized by intense itching, dry skin, eczematous lesions, and epidermal barrier disruption. Its incidence has increased over recent decades, especially in industrialized countries, affecting both children and adults [33]. Its pathophysiology involves a complex interplay among three main factors: a dysfunctional skin barrier, microbial imbalance, and dysregulated immune responses, which mutually reinforce a cycle perpetuating the disease [33]. During acute disease flares, a significant decrease in bacterial diversity is observed together with the overgrowth of *Staphylococcus aureus* within the lesions. This alteration favors inflammation, disrupts barrier function, and amplifies the immune response [34].

Numerous studies have identified genetic alterations in atopic dermatitis patients leading to reduced expression of key structural skin proteins such as filaggrin and claudins. This deficiency compromises the stratum corneum's integrity, promotes transepidermal water loss, and facilitates the penetration of allergens and external microorganisms [20]. As a result, the skin becomes drier and more susceptible, creating an environment conducive to the overproliferation of pathogenic microorganisms such as *S. aureus*, which can be observed even in clinically unaffected skin. During disease exacerbations, *S. aureus* may dominate up to 80%–90% of the microbiome in lesions, displacing commensal species such as *S. epidermidis*, *S. capitis*, or *Micrococcus* spp. [33, 35]. This microbial diversity loss is also associated with reductions in immunomodulatory bacteria like *Lactobacillus*, *Bifidobacterium*, *Propionibacterium*, and *Acinetobacter* [36].

Far from being a passive inhabitant, *S. aureus* actively participates in inflammation by releasing toxins, superantigens, and surface proteins capable of inducing Th2 immune responses. These promote the release of cytokines, such as interleukins (IL) IL‐4, IL‐5, and IL‐13, intensifying inflammation, exacerbating pruritus, and further impairing barrier function, thus perpetuating the inflammatory cycle and facilitating successive colonizations [33, 35].

In summary, current evidence suggests that microbiome imbalance not only accompanies the disease but actively contributes to its development. Consequently, complementary therapies aimed at restoring microbial balance are being considered to address atopic dermatitis more integrally.

*Acne vulgaris* is a chronic inflammatory disease affecting the pilosebaceous unit, particularly prevalent in adolescents and young adults. Its etiology is multifactorial, resulting from the interplay between hormonal changes, alterations in follicular keratinization, dysregulation in the cutaneous microbiota, and an exacerbated immune response [37]. In acne vulgaris, imbalance is manifested as an increase of pr‐inflammatory *C. acnes* strains and a reduction of non‐pathogenic strains that in normal conditions help modulate immune response and sebum production. This intraspecific dysbiosis leads to a wide spectrum of clinical manifestations, ranging from non‐inflammatory comedones to more severe inflammatory lesions, such as papules, pustules, nodules, or cysts, especially in seborrheic areas [37, 38].

The initial event in its development is usually hyperkeratinization of the follicular epithelium, which causes canal obstruction and microcomedone formation, considered the primary lesion. Simultaneously, sebaceous glands increase sebum production under androgen influence, creating an anaerobic environment favorable for the growth of *C. acnes* [39].

**FIGURE 4:**
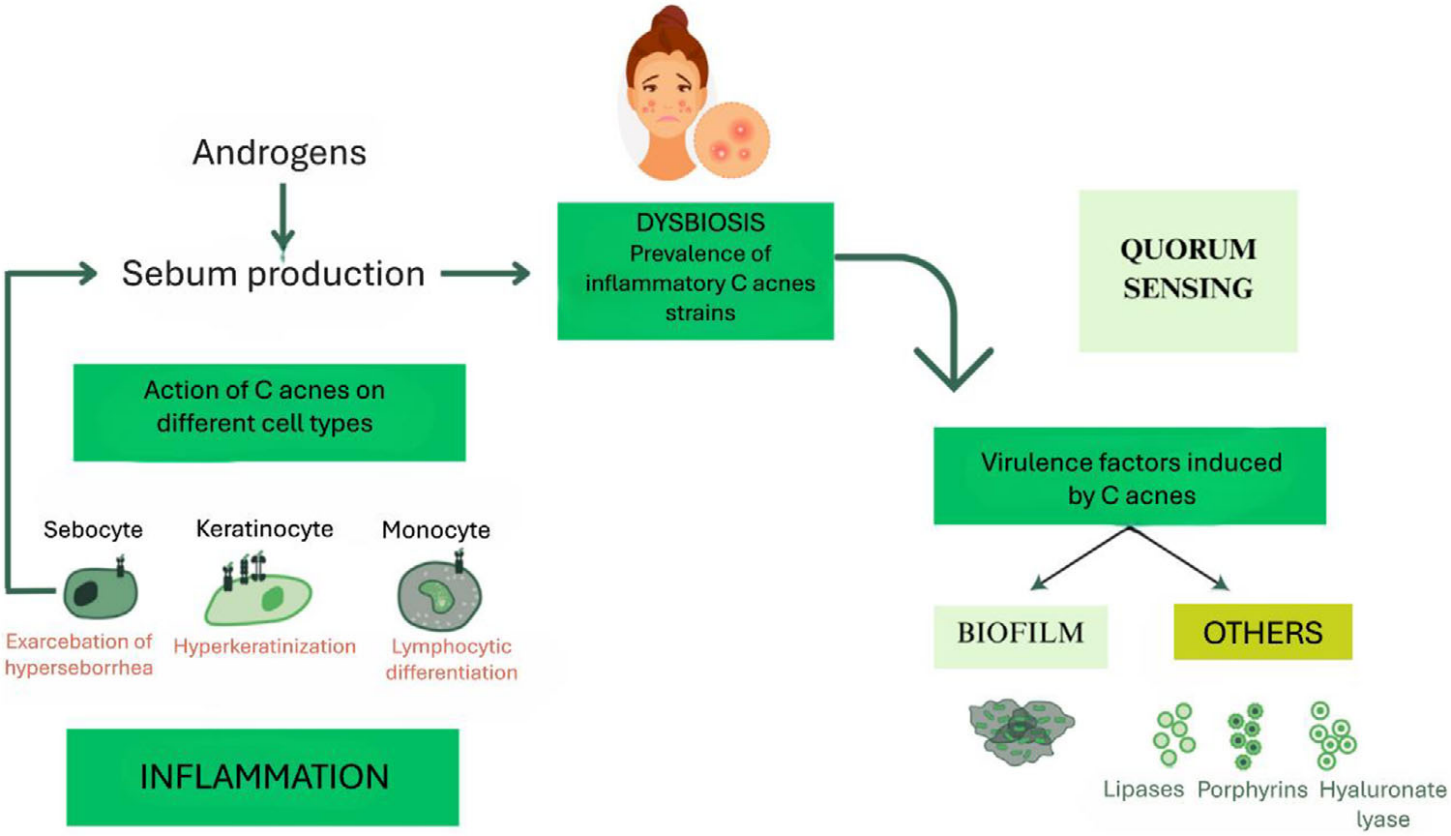
Dysbiosis and inflammation in acne: interaction between androgens, sebum production, and the predominance of virulent *C. acnes* strains. Adapted from [41].

Although *C. acnes* has traditionally been associated with acne pathogenesis, recent studies indicate that, beyond an increased overall bacterial load, a central pathogenic driver is the predominance of specific inflammatory strains, such as the IA1 phylotype, which exert a disproportionate effect on immune activation [40]. These activate immune Toll‐like receptors (TLRs) like TLR2, thereby triggering the production of cytokines such as IL‐1, IL‐8, and TNF‐α [18]. Furthermore, these strains tend to form biofilms and secrete substances such as porphyrins, lipases, and hyaluronate lyase, which contribute to tissue damage and the persistence of lesions [41]. This process is summarized in Figure 4, which schematically represents the pathophysiological cascade of the disease.

This microbial disrupted homeostasis is also accompanied by a reduction in bacterial diversity, including beneficial species such as *S. epidermidis* and *Corynebacterium* spp. In particular, *S. epidermidis* has demonstrated a protective effect by producing succinic acid, which is capable of inhibiting the growth of *C. acnes* and modulating inflammation through the regulation of TLRs and the production of IL‐6 [42].

Another important factor in the persistence of acne is the phenomenon of quorum sensing, a bacterial communication system that regulates the expression of genes related to virulence and biofilm formation. In the context of acne, this mechanism enables *C. acnes* to adapt its behavior by increasing adherence to the follicular epithelium, enhancing resistance to the inflammatory environment, and, in some cases, developing tolerance to topical treatments [41].

Taken together, acne should not be understood as a simple infection, but as a complex alteration of the cutaneous ecosystem, characterized by loss of diversity, imbalance among species, and dominance of inflammatory strains that promote its persistence.

## 4 Impact of Conventional Cosmetics on the Skin Microbiota

In recent years, interest has grown in how the cutaneous microbiota influences skin health, leading to numerous clinical and experimental studies. Cosmetic products represent a key modulating factor of the skin microbiome. Their frequent use may alter or support microbial balance, depending on both their formulation and the compatibility of their ingredients with the skin ecosystem [43].

Conventional cosmetics, typically formulated according to texture, sensorial efficacy, and physical stability, may include ingredients that, although not directly aggressive, affect the viability of beneficial microorganisms [44]. For a long time, it has been thought that some common components in cosmetics, such as preservatives, surfactants, or fragrances, could affect the diversity and balance of the skin microbiome. However, the literature indicates that these effects may depend on several factors, including the composition and concentration of the product, its pH, type of cosmetic formulation, the frequency of use, and the initial condition of the skin [45].

*Preservatives* are used in cosmetic products to prevent the growth of unwanted microorganisms and are responsible for maintaining the appropriate, long‐term quality of cosmetics (in accordance with EU Directive No. 1223/2009) [46]. Nevertheless, they can also influence the beneficial bacteria inhabiting the skin. A representative example is an in vivo human study [43] that analyzed how certain cosmetic formulations containing common preservatives—such as parabens, phenoxyethanol, and isothiazolinones—affected the cutaneous microbiota of healthy women. The study involved the application of “leave‐on” products (such as body lotions) and “rinse‐off” products (such as shower gels) over a 4‐week period. The results showed no significant changes in either alpha or beta diversity of the microbiome. Additionally, the most common bacteria, including *Cutibacterium*, *Staphylococcus*, and *Corynebacterium* maintained their usual levels. The formulations were well tolerated, and no adverse effects were reported. Thus, commonly used preservatives in cosmetics do not appear to significantly alter the skin microbiota, provided they are formulated at appropriate concentrations.

*Surfactants*, found in products like soaps, gels, or shampoos, act as cleansing agents due to their molecular structure, which includes a hydrophilic (water‐attracting) part and a hydrophobic (oil‐repelling) part. This allows them to form micelles that encapsulate sebum, dirt, and other impurities, facilitating their removal upon rinsing with water. Although highly effective at cleansing the skin, surfactants can also affect the lipids and proteins of the stratum corneum, potentially leading to swelling, protein denaturation, and decreased cohesion among cells, thereby compromising the skin barrier function. The degree of irritation caused by these compounds varies according to their chemical structure and the concentration used. Therefore, selecting appropriate surfactants is essential, especially in formulations designed for sensitive skin [45].

*Emollients*, such as vegetable oils, shea butter, or ceramides, together with occlusive agents like petrolatum, help preserve skin hydration and reinforce barrier function. Generally, their impact on the microbiota is neutral or even beneficial. In fact, a clinical study [47] in women with dry skin demonstrated that the use of a lotion formulated with glycerin, free fatty acids, and small amounts of occlusives significantly improved hydration, increased levels of essential stratum corneum lipids such as ceramides, cholesterol, and long‐chain fatty acids, and promoted greater cohesion on the skin surface. Regarding the microbiota, a specific increase in *S. epidermidis* was recorded without changes in overall diversity. Additionally, an improvement in microbial functionality was observed, highlighting increased production of beneficial lipids such as palmitate, oleate, and stearate [47]. Ultimately, these results reflect how certain topical formulations can not only benefit skin hydration and structure but also positively modulate the cutaneous microbiota.

Many conventional cosmetic products also include ingredients with moisturizing, regenerative, and soothing effects, such as *vitamins*, *provitamins*, and *bioactive agents*. One study evaluated a basic facial care routine containing dexpanthenol (provitamin B5), niacinamide, and glycerin. After 4 weeks of use, participants showed visible improvements in hydration, elasticity, and barrier function, with no significant changes detected in overall microbiome composition or diversity in this study [48]. These findings suggest that such functional ingredients may provide dermatological benefits without overtly disturbing the skin's microbial balance under the tested conditions; however, broader and longer‐term studies are needed to confirm that even subtle, non‐dysbiotic shifts have no clinically relevant impact.

The *pH* of cosmetic products plays a key role in maintaining the balance of the skin microbiota. A slightly acidic pH, between 4.5 and 5.5, creates a favorable environment for beneficial skin bacteria, whereas more alkaline values can disrupt this balance and promote the onset of dysbiosis. In a randomized clinical trial [21], products with a pH below 5, including vitamin C, resveratrol, and collagen and algae masks, were evaluated. Use of these formulations reduced the presence of *Corynebacterium*, a bacterium associated with microbial imbalances, without affecting protective species such as *S. epidermidis*. Furthermore, an improvement in the skin's barrier function was observed. Complementarily, another clinical trial [49] compared two “leave‐on” lotions with identical composition but different pH values (5.5 vs. 9.3), applied after acute skin insult. Although both promoted recovery of the microbiota and barrier function, the lotion with acidic pH showed faster recovery. This underscores the role of pH as a protective factor.

Overall, current evidence indicates that many conventional formulations, when appropriately designed in terms of concentration, pH and regimen, do not induce major dysbiosis in healthy skin. Nonetheless, the possibility that small, subclinical alterations in community structure or function may accumulate over time warrants cautious interpretation of existing data and further longitudinal, multi‐omic investigations.

## 5 Microbiome‐Friendly Cosmetics for Skin Balance

In the field of scientific cosmetics, the concept of microbiome‐friendly products has become increasingly common to identify formulations that preserve the diversity and stability of the skin ecosystem. Innovation in this area has led to the incorporation of active ingredients that directly modulate the microbiome, each with specific properties and benefits (Table 3). Hence, these products are developed with the objective of maintaining, restoring, or beneficially influencing the cutaneous microbiota without compromising its diversity or composition [44, 49].

**TABLE 3:** Microbiome‐friendly cosmetic ingredients.

Active ingredient	Description	Main functions	Reference
Probiotics	Live microorganisms applied topically to the skin.	Balance the microbiome, strengthen the skin barrier, and modulate inflammation.	[44]
Prebiotics	Compounds that serve as substrates for beneficial bacteria.	Stimulate the growth of protective skin bacteria.	[51]
Postbiotics	Bioactive compounds derived from microbial metabolism (organic acids, peptides, lipids).	Reinforce the skin barrier, reduce inflammation, and improve hydration—without the risks associated with introducing live microorganisms.	[52, 53]
Paraprobiotics	Inactive microorganisms or cell lysates that do not require viability to exert beneficial effects.	Provide beneficial effects similar to probiotics, with improved safety and stability.	[53, 54]

Beyond this general framework, it is important to distinguish microbiome‑friendly formulations, primarily designed to be safe, non‑disruptive, and supportive of the existing microbial equilibrium, from genuinely microbiome‑targeted strategies that pursue active modulation of community structure and function.

In current cosmetic innovation, the focus is progressively shifting from mere preservation or neutrality toward interventions explicitly aimed at correcting dysbiosis, enriching beneficial taxa, or attenuating pathogenic overgrowth through probiotic, prebiotic, postbiotic, paraprobiotic, and related bioactive approaches. From a technical standpoint, this evolution entails moving from formulations that simply avoid perturbing microbial diversity to those intentionally engineered to remodel microbial networks, signaling pathways, and barrier‑associated processes, thereby positioning microbiome‑targeted cosmetics as potential adjuvants in the management of microbiome‑associated skin disorders [50]. Critically, while promising, microbiome‐friendly claims lack harmonized ISO/USP criteria and require large‐scale randomized controlled trials for clinical validation.

The analysis of cosmetic products containing probiotics, prebiotics, postbiotics, and other functional compounds has allowed to demonstrate visible benefits such as improved hydration or wrinkle reduction, significant modifications in the composition and balance of the cutaneous microbial ecosystem. In fact, these ingredients represent the primary innovative strategies to modulate the skin's microbiome through cosmetics as described below.

Topically applied *probiotics* are live microorganisms capable of beneficially influencing the balance of the skin microbiome. Numerous studies support their efficacy in improving various clinical and microbiological parameters [55, 56, 57, 58].

A prominent example is *Micrococcus luteus* Q24, a commensal strain analyzed for its safety, efficacy, and ability to colonize the skin post‐application. Two recent studies evaluated its cosmetic potential. In the first, involving 47 adults, different formulations (cream and serum) were well tolerated, promoted hydration, improved skin texture, and reduced potentially pathogenic staphylococci [58]. In a second study with 10 women over 25 days, the authors reported, under the assay conditions, notable improvements in hydration and skin appearance (reductions in pores, wrinkles, spots, and impurities), without interfering with the native microbiome. These benefits are attributed to the strain's ability to form a protective microbial barrier, exert antioxidant action, and inhibit enzymes related to skin aging [57]. However, it should be noted that small sample size, short duration and limited methodological details (e.g. blinding, statistical power) make it difficult to fully assess the robustness and reproducibility of these findings. Therefore, although these products have shown *initial* clinical and microbiological benefits in selected populations, further confirmation is required. Also noteworthy is *C. acnes* subsp. *defendens* XYCM42, a strain recently described in a clinical study [59] with antioxidant, anti‐inflammatory, and immunomodulatory properties. It has been shown to stimulate key genes involved in skin barrier function and collagen synthesis, while reducing proinflammatory cytokine expression. Microbiologically, it successfully established controlled colonization on the skin, decreasing the relative proportion of *Staphylococcus* spp. without negatively affecting microbiome diversity. The study included an 8‐week clinical trial with 121 participants, assessing a cosmetic routine based on the live strain, its fermentate, and products designed to enhance its implantation and functionality on the skin. The routine was divided into two daily phases: a morning routine including a gentle prebiotic cleanser, the serum with the fermentate, a moisturizer adapted to skin type, and a sunscreen; and a nighttime routine, starting with the same cleanser followed by a gel containing live XYCM42 cells and a prebiotic activator formulated with compounds that enhance the strain's activity. This comprehensive approach established an optimal environment for XYCM42's action, resulting in visible improvements in hydration, elasticity, sebum control, reduction of erythema, wrinkles, spots, and skin texture—all without adverse effects or negative alteration of cutaneous microbiome balance [59].

These limitations arise from the technological challenges related to the direct incorporation of live probiotic bacteria into cosmetic formulations. To address these challenges and enable the use of probiotic as an active ingredient in cosmetics, alginate microspheres were used as a carrier system.

In conventional cosmetics, the inclusion of live probiotic bacteria is limited by the need for preservatives, as required by EU Directive [46]. This restriction appears from the technological difficulties of integrating viable probiotics into cosmetic formulations. To overcome these issues and allow their use as active ingredients, alginate microspheres, for example, can be used as a carrier system [60, 61].

*Prebiotics*, such as certain plant‐derived oligosaccharides, fructooligosaccharides (FOS), galactooligosaccharides (GOS), and specific botanical extracts, do not contain live microorganisms but rather substances that selectively promote the growth of beneficial bacteria already present on the skin [62]. In this regard, a randomized, double‐blind, placebo‐controlled clinical trial evaluated the use of a facial serum containing GOS in a group of 60 women aged 40–60 years over 8t weeks [62]. The results showed a remarkable improvement in skin hydration, as well as a decrease in transepidermal water loss and erythema. At the microbiome level, an increase in microbial diversity was detected, with a predominance of beneficial genera such as *Pediococcus* and *Lactococcus*, and a significant reduction of *S. aureus*. No adverse effects or signs of dysbiosis were reported, supporting the safety and effectiveness of topical GOS use as a prebiotic strategy to strengthen the skin barrier and improve the condition of mature skin.

Furthermore, beyond the separate use of probiotics or prebiotics, *“symbiotic” formulations*, which combine both components, have demonstrated positive effects clinically and microbiologically. An innovative example is that of symbiotic baths, studied in a randomized clinical trial involving 22 individuals with atopic dermatitis [36]. The intervention combined live strains of *Lactobacillus* and *Bifidobacterium* with prebiotics such as inulin, maltodextrin, and apple pectin. Participants treated with this formulation showed significant improvements in the SCORAD index, a clinical tool that evaluates the severity of atopic dermatitis by combining the extent and intensity of lesions with subjective symptoms such as pruritus. A decrease in this index indicates overall improvement. Moreover, specific ameliorations were observed in pruritus, redness, dryness, and lichenification, without adverse effects. Microbiologically, effective colonization of probiotics on the treated skin was achieved, alongside a reduction of proinflammatory genera such as *Corynebacterium* and *Brevibacterium*, and greater microbiome stability [36].

In recent years, *postbiotic* **s** have become an increasingly utilized option within dermocosmetics, offering benefits similar to live probiotics but with greater stability and safety. These compounds do not contain live microorganisms but consist of substances generated during microbial fermentation, such as metabolites or cellular fragments, which exert positive effects on the skin. Among their most notable properties are anti‐inflammatory, antioxidant, and immunoregulatory capacities, making them effective allies for restoring cutaneous microbiota balance without the drawbacks associated with maintaining live bacteria [38].

One major advantage of postbiotics is their ease of incorporation into cosmetic formulations, as they do not require refrigeration nor carry a high risk of eliciting immune reactions. A notable example is butyric acid, a compound produced by bacteria such as *S. epidermidis*, extensively studied for its skin benefits. However, its topical use has been limited by its strong odor and unpleasant texture. To overcome this issue, phenylalanine butyramide (FBA), a solid, odorless derivative that gradually releases butyric acid on the skin, was developed. This derivative has been observed to have a remarkable calming and anti‐redness effect, reducing erythema by up to 17.8% one hour post‐application without altering the skin barrier. This effect is attributed to activation of cellular receptors such as GPR43, involved in inflammation regulation [63].

In another study, two types of emollients were compared for atopic dermatitis treatment: a conventional formula and one enriched with prebiotic and postbiotic ingredients [64]. The study included 54 adults with mild to moderate atopic dermatitis, who applied both products on different arms for four weeks. The enriched emollient contained La Roche‐Posay thermal water, niacinamide, and biomass from *Vitreoscilla filiformis*, all known for beneficial effects on microbiota and skin barrier function. Although both products helped improve symptoms, the enriched emollient was more effective in reducing *S. aureus* presence, accelerating barrier recovery, and notably decreasing redness. Moreover, it achieved a significantly greater reduction in itch intensity from the second week of application, according to the visual analog scale used in the study [64].

Additionally, a double‐blind clinical trial evaluated the efficacy and tolerability of a topical gel applied to individuals with mild to moderate acne [39]. This gel contained lactic acid, modified azelaic acid, azelamidopropyl dimethylamine, and *Bifida* ferment lysate, a postbiotic ingredient capable of modulating skin pH and microbiota. Sixty subjects were randomly assigned to a treatment group (*n* = 30) or a placebo group (*n* = 30), applying the product twice daily for 8 weeks. The treated group showed a significant improvement in lesion count, inflammation, and sebum production, with no reported adverse effects.

Finally, the concept of *“triple‐biotic” products*, which combine probiotics, prebiotics, and postbiotics, represents an evolution in cosmetic care. An interesting study utilized advanced techniques such as microbiome sequencing and metabolome analysis to evaluate a regimen based on ingredients like inulin, lactic acid, and pyruvic acid [65]. Results of this study showed an increase in *S. epidermidis*, reduction of *Pseudomonas stutzeri*, and improvements in both microbial diversity and skin hydration and barrier integrity.

In summary, postbiotics are gaining prominence in the cosmetic field due to their proven clinical benefits, both when used alone and as part of more comprehensive formulations. Their versatility and efficacy position them as a promising tool to treat and care for various skin types.

Among emerging cosmetic ingredients, *paraprobiotics*, heat‐inactivated bacteria, are establishing themselves as a safe and functional alternative [53]. Although not alive, they retain the ability to interact with the immune system and keratinocytes without the risks associated with active microorganisms [54].

In this context, *Lactobacillus plantarum* has garnered attention for its positive effects on skin. Two recent studies examined its use from distinct yet complementary perspectives. The first one, conducted with 15 women over two months, analyzed a facial cream formulated with heat‐inactivated *L. plantarum* GMNL6 [54]. Results showed notable improvements in hydration, pigmentation, redness, and overall skin appearance. Additionally, favorable changes in the skin microbiota were observed, with a reduction in *Cutibacterium* and an increase in genera such as *Streptococcus* and *Staphylococcus*, considered beneficial for skin health.

The second study, with a larger sample of 50 individuals, evaluated a moisturizing formula combining heat‐inactivated *L. plantarum* and *Bifidobacterium lactis* [66]. After 4 weeks of application, improvements in skin hydration levels and reductions in potentially harmful microorganisms such as *Cutibacterium*, *Corynebacterium*, and *Acinetobacter* were recorded.

Both studies concur that heat‐inactivated *L. plantarum* can confer benefits at molecular levels as well as through its influence on the skin's microbial ecosystem.

Separately, another clinical investigation evaluated the cosmetic use of *Pediococcus acidilactici* PMC48, a strain isolated from sesame leaf kimchi, targeting hyperpigmentation treatment [67]. Unlike other treatments which only inhibit melanin formation, this strain was capable of directly degrading existing melanin and inhibiting tyrosinase, similarly to compounds like arbutin. During an 8‐week trial with 22 volunteers, a tonic containing 2.5% culture supernatant was applied on UV‐induced hyperpigmented areas. Significant improvements were observed: a 47.6% reduction in color intensity, an 8.1% increase in brightness, an 11.8% decrease in melanin index, and a 20.9% increase in hydration. Additionally, 16S rRNA sequencing analysis of the microbiota showed a significant increase in *Lactobacillaceae* without negative effects on other bacteria, reinforcing its safety profile.

Together, these studies highlight the promising role of paraprobiotics as effective cosmetic ingredients capable of improving skin health by regulating microbiota, hydration, and pigmentation, even in the absence of viable organisms.

All these considerations (summarized in Table 4) underscore the importance of a rational design of formulation, where ingredients are selected not only for their cosmetic efficacy, but also for their ecological impact on the skin ecosystem. In this regard, microbiome‐friendly cosmetics could represent an innovative and promising strategy to preserve or restore the balance of the skin microbiome, thereby promoting skin health and preventing states of dysbiosis that may lead to inflammatory diseases.

**TABLE 4:** Studies on the impact of cosmetic products on the skin microbiota and skin health.

Study type	Sample/population	Intervention	Main findings	Reference
Clinical study	15 Women (25–50 years)	Facial cream with *Lactobacillus plantarum* GMNL6 (inactive bacterium)	Improved hydration, wrinkles, spots, texture, skin microbiome, and collagen synthesis	[54]
Double‐blind clinical trial	22 Patients with atopic dermatitis	Daily baths for 14 days: (1) symbiotic; (2) prebiotics only; (3) placebo	Significant SCORAD reduction with symbiotic and prebiotics; pruritus and dryness improved only with symbiotic; increased colonization by beneficial skin bacteria	[36]
Self‐controlled clinical study	20 Patients (12 women, 3 men) with mild to moderate acne	Topical postbiotic + microneedling	Significant acne improvement, high satisfaction, no major adverse effects	[38]
Randomized, double‐blind, placebo‐controlled clinical trial	60 Patients with mild to moderate acne (16 years old)	Topical gel with lactic acid, azelaic acid, azelamidopropyl dimethylamine, and *Bifida* lysate, twice daily for 2 months	Significant lesion improvement vs. placebo; no adverse effects	[39]
Longitudinal study	37 Women (18–55 years) with dry skin	Body lotion applied twice daily	Improved hydration, increased skin lipids, increased *S. epidermidis*	[43]
Crossover clinical trial	50 Subjects with atopic dermatitis	BSD and DAC creams, 3 months each	Improved hydration and symptoms; no changes in microbiome	[48]
In vivo experimental study	25 Healthy adults	Application of lotions at pH 5.5 and 9.3	Both lotions accelerated microbiome and barrier function recovery; better effect with pH 5.5	[49]
Open cosmetic study	10 Healthy women (18–60 years)	Serum with *Micrococcus luteus* Q24, twice daily for 25 days	Improved hydration (+101%), reduced pores, wrinkles, spots, and impurities	[55]
Preliminary controlled, single‐blind trial	47 Healthy adults (18–75 years)	Topical application of *M. luteus* Q24 in cream or serum	Safe and well tolerated; improved skin appearance and good probiotic colonization	[55]
Clinical pilot and main study	10 (Pilot) and 121 (main) subjects	Topical application of *Cutibacterium acnes* XYCM42 and ferment	Improved hydration, texture, tone, sebum, and erythema; no adverse events	[59]
Randomized clinical trial	60 Women (40–60 years)	Facial serum with galacto‐oligosaccharides (GOS), 8 weeks	Improved hydration, wrinkle reduction, decreased *S. aureus*, increased microbial diversity	[62]
In vivo experimental study	20 Healthy volunteers (20–60 years)	Emulsion with 1.5% fermented barley extract (FBA) after induced erythema	Reduced erythema; soothing effect	[63]
Randomized clinical trial	57 Adults with moderate–severe atopic dermatitis	Emollient plus + syndet vs. usual care	Reduced pruritus, improved quality of life, and decreased use of emollient plus products	[64]
Multi‐omics randomized clinical study	53 Women with dry or very dry skin	Bath gel and lotion with or without pre/postbiotics for 6 weeks	Increased hydration in both groups; pre/postbiotic group showed reduction of pathogenic bacteria, increased commensals, and beneficial metabolome changes	[65]
Preliminary controlled study	50 Healthy adults (25 treatment, 25 control)	Cream with paraprobiotics (*Bifidobacterium lactis* and *Lactobacillus plantarum*)	Improved skin hydration and positive changes in the cutaneous microbiome	[66]
Clinical trial	22 Korean women	Topical application of *Pediococcus acidilactici* PMC48 for 8 weeks	Decreased melanin, increased brightness and hydration; no adverse effects	[67]

It is noteworthy that despite the remarkable advances in characterizing the skin microbiome, several bottlenecks still hinder translating this knowledge into microbiome‐based cosmetic development. The main limitations include methodological aspects, such as the low microbial biomass of skin samples, and the lack of reproducible in vitro models that accurately reflect the complexity and dynamics of the skin ecosystem. Moreover, the absence of standardized protocols that would allow reliable comparison across studies. In addition, most of the available clinical evidence on microbiome‑friendly cosmetics comes from small, short‑term, often open or single‑blind studies with heterogeneous outcome measures and not always including double‑blind, placebo‑controlled conditions. Thus, another critical limitation lies in the scarcity of clinical evidence validating the mechanisms of action and efficacy of microbiome‐cosmetic ingredients, coupled with the absence of harmonized regulatory frameworks. Consequently, current data on microbiome‑targeted ingredients such as probiotics, prebiotics, postbiotics, and paraprobiotics should be regarded as promising but still exploratory and cannot yet be extrapolated to all cosmetic formulations within this category. There is a clear need for large‑scale, rigorously designed randomized controlled trials with standardized clinical and microbiological endpoints, long‑term follow‑up coupled with harmonized regulatory frameworks before the clinical impact and mechanisms of these microbiome‑friendly formulations can be considered fully established and translated into robust, reproducible cosmetic innovations. Overall, addressing these methodological and regulatory challenges is essential to develop microbiome‐friendly cosmetics that support a more functional, personalized, and dynamic understanding of the skin microbiome to improve human health. In addition, by promoting skin‐care strategies that support cutaneous health while encouraging more informed and responsible use of cosmetic formulations.

## 6 Conclusion

The skin is colonized by a highly diverse ecosystem of microorganisms, with commensal bacteria such as *C. acnes*, *S. epidermidis*, and various *Corynebacterium* species playing key roles in maintaining cutaneous homeostasis. Endogenous factors, including age, genetics, gender, hormonal status, and exogenous factors influence such as climate, hygiene, cosmetics, and topical or systemic treatments, dynamically modulate the composition and diversity of the cutaneous microbiome. These factors can compromise the integrity of the skin barrier and reduce microbial diversity, increasing susceptibility to microbial imbalance or dysbiosis, thereby contributing to inflammatory diseases such as atopic dermatitis and acne. Conventional cosmetics may either support or disturb the skin microbiota, with their impact largely dependent on formulation, pH, concentration and application regimen. Although current data suggest that appropriately designed products can improve skin barrier function without causing obvious dysbiosis, small shifts in the microbiome could have long‑term consequences that are not yet fully understood. Hence, the design and selection of skincare and cosmetic innovative products should prioritize the preservation of microbial balance as a key strategy for maintaining healthy, resilient skin and preventing the onset or exacerbation of dermatological disorders. Consequently, a more refined dermatological approach that respects and preserves the native microbiota is both necessary and timely. In this context, early clinical data indicate that microbiome‐friendly cosmetics, especially those enriched with probiotics, prebiotics, postbiotics, or paraprobiotics, have clinical and microbiological benefits, including the promotion of microbial diversity and barrier function, without adverse effects and represent a promising strategy for enhancing skin health and managing cutaneous dysbiosis. In this sense, microbiome‑targeted cosmetics should be viewed as an encouraging yet still preliminary approach that must be integrated with scientific caution and rigorous, evidence‑based claims.

## Conflicts of Interest

The authors declare no conflicts of interest.

## Data Availability

Data sharing not applicable to this article as no datasets were generated or analyzed during the current study.
